# Imbalanced motivated behaviors according to motor sign asymmetry in drug-naïve Parkinson’s disease

**DOI:** 10.1038/s41598-023-48188-0

**Published:** 2023-12-01

**Authors:** Matthieu Béreau, Anna Castrioto, Mathieu Servant, Eugénie Lhommée, Maxime Desmarets, Amélie Bichon, Pierre Pélissier, Emmanuelle Schmitt, Hélène Klinger, Nadine Longato, Clélie Phillipps, Thomas Wirth, Valérie Fraix, Isabelle Benatru, Franck Durif, Jean-Philippe Azulay, Elena Moro, Emmanuel Broussolle, Stéphane Thobois, Christine Tranchant, Paul Krack, Mathieu Anheim

**Affiliations:** 1 Neurology Department, University Hospital of Besançon, CHRU de Besançon, 3 Bd Alexandre Fleming, 25030 Besançon Cedex, France; 2 Laboratoire de Recherches Intégratives en Neurosciences et Psychologie Cognitive - UR LINC, Université Bourgogne Franche-Comté, Besançon, France; 3 Inserm, U1216, Grenoble Institut Neurosciences, CHU Grenoble Alpes, University Grenoble Alpes, 38000 Grenoble, France; 4 Unité de Méthodologie, CIC INSERM 1431, CHU de Besançon, Besançon, France; 5 Movement Disorders Unit, Neurology Department, Hospices Civils de Lyon, Lyon, France; 6 Faculté de Médecine Lyon Sud, Université Claude Bernard Lyon 1, University of Lyon, Lyon, France; 7 CNRS, Institut des Sciences Cognitives Marc Jeannerod, UMR 5229, Bron, France; 8 Service de Neurologie, Hôpitaux Universitaires de Strasbourg, Strasbourg, France; 9 Institut de Génétique et de Biologie Moléculaire et Cellulaire (IGBMC), INSERM-U964/CNRS-UMR7104, Université de Strasbourg, Illkirch, France; 10 Fédération de Médecine Translationnelle de Strasbourg (FMTS), Université de Strasbourg, Strasbourg, France; 11 Neurology Department, University Hospital of Poitiers, Poitiers, France; 12 INSERM, CHU de Poitiers, Centre d’Investigation Clinique CIC1402, University of Poitiers, Poitiers, France; 13 EA7280 NPsy-Sydo, Université Clermont Auvergne, Clermont-Ferrand, France; 14 Neurology Department, Clermont-Ferrand University Hospital, Clermont-Ferrand, France; 15 Movement Disorders Unit, Neurology Department, University Hospital of Marseille, Marseille, France; 16 Department of Neurology, Movement Disorders Center, University Hospital of Bern, Bern, Switzerland

**Keywords:** Neurology, Parkinson's disease

## Abstract

Few studies have considered the influence of motor sign asymmetry on motivated behaviors in de novo drug-naïve Parkinson’s disease (PD). We tested whether motor sign asymmetry could be associated with different motivated behavior patterns in de novo drug-naïve PD. We performed a cross-sectional study in 128 de novo drug-naïve PD patients and used the Ardouin Scale of Behavior in Parkinson’s disease (ASBPD) to assess a set of motivated behaviors. We assessed motor asymmetry based on (i) side of motor onset and (ii) MDS-UPDRS motor score, then we compared right hemibody Parkinson’s disease to left hemibody Parkinson’s disease. According to the MDS-UPDRS motor score, patients with de novo right hemibody PD had significantly lower frequency of approach behaviors (*p* = 0.031), including nocturnal hyperactivity (*p* = 0.040), eating behavior (*p* = 0.040), creativity (*p* = 0.040), and excess of motivation (*p* = 0.017) than patients with de novo left hemibody PD. Patients with de novo left hemibody PD did not significantly differ from those with de novo right hemibody PD regarding avoidance behaviors including apathy, anxiety and depression. Our findings suggest that motor sign asymmetry may be associated with an imbalance between motivated behaviors in de novo drug-naïve Parkinson’s disease.

## Introduction

Parkinson’s disease (PD) is a neuropsychiatric condition that combines a broad range of motor and non-motor signs, even in the early stages of the disease^1–3^. Among non-motor signs, behavioral syndromes including apathy and impulse control disorders (ICD) are frequently encountered and have a substantial impact on patient and caregiver quality of life^4–6^. These behavioral disorders result from a complex interplay between dopaminergic denervation within nigrostriatal and mesocorticolimbic pathways, dopamine replacement therapy (DRT), and limbic and executive fronto-striatal circuits^4,6^.

Some clinical and neurophysiological studies in healthy subjects or patients with brain diseases have reported frontal lobe lateralization in reward and punishment processing and motivated behaviors^7–10^. Overall, these studies demonstrated greater activation of the left prefrontal cortex in reward processing and approach behaviors and greater activation of the right prefrontal cortex in punishment processing and avoidance behaviors^7–10^. Most evidence comes from unilateral stroke lesions, which have demonstrated approach behaviors including inappropriate euphoria or mania in cases of right frontal damage and avoidance behaviors including depression or the so called “catastrophic reaction” in cases of left hemisphere lesions^11,12^. Importantly, authors have assumed that avoidance and approach behaviors are inversely related to each other, such that loss of one motivated behavior may lead to an imbalance in favor of the other^11^. This hypothesis is also supported by previous studies in healthy subjects and patients with behavioral-variant frontotemporal dementia (bv-FTD), which have shown better approach learning in healthy subjects with larger reward responses in the left ventral striatum, and behavioral disinhibition in bv-FTD patients with right-sided asymmetric orbitofrontal grey matter pathology^13,14^.

PD is characterized by asymmetric motor symptoms, which reflect asymmetric loss of dopaminergic neurons within motor circuits^15–18^. It has therefore been hypothesized that asymmetric dopaminergic denervation within non-motor fronto-striatal circuits in PD may contribute to different patterns of motivated behaviors^19^. Previous studies that have examined the relationship between side of onset of motor signs and the occurrence of motivated behavior disorders in PD have reported conflicting results^20–23^. Disease duration and dopamine replacement therapy (DRT) may also have been confounding factors. Moreover, two studies in de novo PD failed to demonstrate the influence of motor sign asymmetry on the occurrence of behavioral manifestations^19,24^. These discrepancies may be related to the definition of PD asymmetry, the heterogeneity of PD populations and the size of the sample. We present a cross-sectional study of 128 patients with drug-naïve de novo PD, aiming to examine various aspects of motivated behaviors to test whether motor sign asymmetry is associated with an imbalance between approach and avoidance behaviors.

## Materials and methods

### Patient population

We included patients diagnosed with PD according to UK Brain Bank criteria for less than two years without significant cognitive impairment, defined as a score on the Mattis dementia rating scale (MDRS) > 130/144 or on the frontal assessment battery (FAB) > 15/18)^25^. We excluded patients undergoing treatment with levodopa and/or dopamine agonists, as well as MAO-B inhibitors, and/or psychotropic drugs including anxiolytics and antidepressants^25^.

### Study design

This cross-sectional study was ancillary to the “Honeymoon” study, a French prospective multicenter trial. Detailed methodology of the “Honeymoon” study has been described elsewhere^25^.

### PD asymmetry

Right hemibody PD (RPD) and left hemibody PD (LPD) were distinguished based on (i) the declarative hemibody side of onset of motor symptoms^23^; (ii) the lateralized items of the Movement Disorder Society-Unified Parkinson’s Disease Rating Scale (MDS-UPDRS) part III (items 3b–e, 4–8, 15–16, 17a–d) at the time of examination^26^. We calculated a laterality index adapted from Foster et al*.* using the following formula: 2 × (MDS-UPDRS right − MDS-UPDRS left)/(MDS-UPDRS right + MDS-UPDRS left)^27^. A score within the range [-2; 0[ indicated LPD whereas a score within the range ]0; 2] indicated RPD^27^. Participants with indeterminable right or left hemibody PD were excluded from the corresponding analyses.]0;2]

### Clinical assessment

We used the Ardouin Scale of Behavior in Parkinson’s Disease (ASBPD) to assess the whole behavioral spectrum of PD^28^. The ASBPD is a semi-structured clinical interview in which trained psychologist assesses the severity of each hypodopaminergic and hyperdopaminergic item. It consists of 21 items, each of which are rated from 0 (no change) to 4 (severe change). For hypodopaminergic items, we considered scores ≥ 2, which indicate a moderate behavioral impairment, as clinically relevant in early-stage unmedicated PD patients^29^. We added the number of items with a score ≥ 2 for apathy, anxiety and depression to calculate the avoidance behavior composite score (AvCS). For hyperdopaminergic items, de novo unmedicated PD patients are unlikely to exhibit ICD, which appears during progression of PD with chronic exposure to DRT, especially dopamine agonists^30^. We therefore considered scores ≥ 1, which indicates slight but clinically significant behavioral impairment, as the relevant cut-off to account for motivational imbalance in drug-naïve PD patients. We added together the number of items with a score ≥ 1 for nocturnal hyperactivity, eating behavior, creativity, hobbyism, punding, risk taking behavior, compulsive shopping, pathological gambling, hypersexuality and excess in motivation to calculate the approach composite score (AppCS)^23^. All patients underwent neuropsychological assessment including global cognitive efficiency and executive function using the MDRS and the FAB, respectively.

### Statistical analyses

We compared the LPD and RPD groups using the chi-squared test for categorical variables and the Student’s t- test for quantitative variables. All statistical tests were two sided with a significance threshold of 0.05.

### Ethical approval

 The “Honeymoon” study was approved by the Ethics Committee of Grenoble, authorized by the National Agency for the Safety of Medicines and Health Products (AFSSAPS), and registered as NCT02786667. All patients included in the study gave written informed consent in accordance with local legislation. All methods were performed in accordance with the relevant guidelines and regulations.

## Results

**Figure 1:**
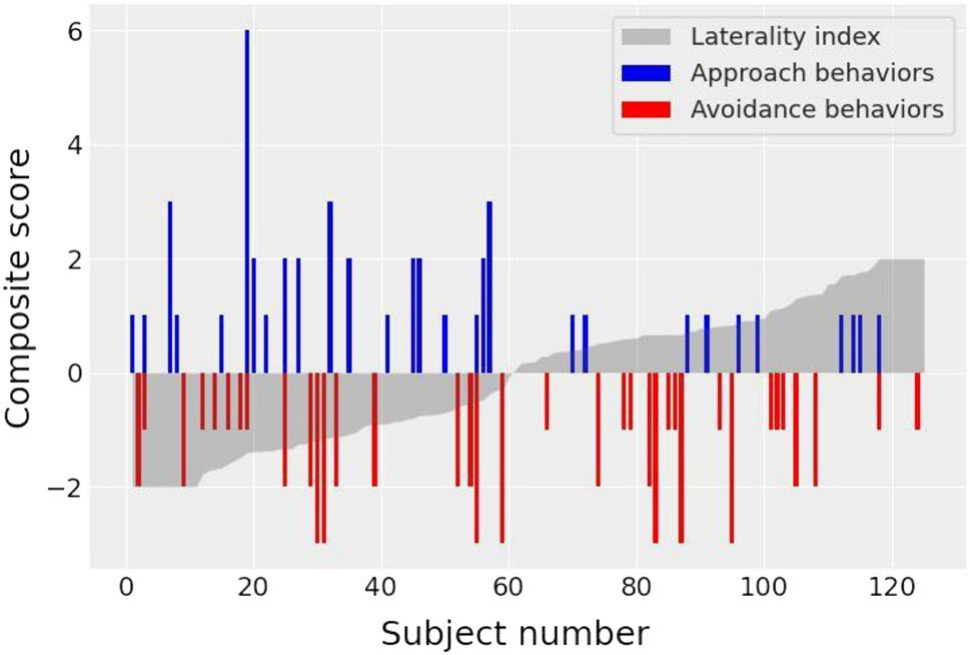
Approach and avoidance composite scores according to laterality index in de novo unmedicated Parkinson’s disease (PD). From left to right, patients are ranked from the lowest (LPD) to the highest (RPD) laterality index score. LPD: left hemibody onset PD, RPD: right hemibody onset PD.

Patient characteristics are described in detail in Table 1 and the relationship between laterality index, AppCS and AvCS is presented in Fig. 1.

**Table 1:** Patient characteristics.

	Laterality based on side of onset (N = 124*)			Laterality based on MDS-UPDRS III (N = 125*)		
	LPD (n = 58)	RPD (n = 66)	p value	LPD (n = 60)	RPD (n = 65)	p value
Socio demographic variables						
Mean age (years, mean, SD)	58.5 (8.1)	59.1 (9.8)	0.702	58.5 (8.5)	59.3 (9.6)	0.661
Disease duration (years, mean, SD)	2.2 (1.5)	2.3 (1.3)	0.777	2.3 (1.4)	2.2 (1.2)	0.583
Sex (male/female)	35/23	45/21	0.363	35/25	45/20	0.205
Motor (mean, SD)						
UPDRS-III total score	23.8 (9.4)	25.2 (9.6)	0.412	25.1 (9.6)	24.3 (9.6)	0.659
UPDRS-III left subscore	13.2 (5.4)	6.1 (5.2)	< 0.001	14.4 (4.8)	5.0 (3.8)	< 0.001
UPDRS-III right subscore	4.5 (4.3)	12.7 (5.5)	< 0.001	4.3 (4.0)	13.1 (5.2)	< 0.001
Cognition (mean, SD)						
MDRS	139.1 (3.4)	139.3 (3.4)	0.784	139.2 (3.1)	139.0 (3.7)	0.722
FAB	16.7 (1.0)	16.7 (1.1)	0.841	16.7 (0.9)	16.7 (1.1)	0.761
Avoidance behaviors (n ≥ 2,%)						
Avoidance behavior composite score	20 (34.5)	18 (27.3)	0.385	18 (30.0)	18 (27.7)	0.776
Depressed mood	10 (17.2)	7 (10.6)	0.284	10 (16.7)	7 (10.8)	0.337
Anxiety	16 (27.6)	10 (15.1)	0.090	14 (23.3)	10 (15.4)	0.260
Irritability, agressiveness	2 (3.4)	1 (1.5)	0.493	2 (3.3)	1 (1.6)	0.521
Hyperemotivity	10 (17.2)	10 (15.1)	0.752	8 (13.3)	11 (16.9)	0.576
Apathy	12 (20.7)	10 (15.1)	0.420	10 (16.7)	11 (16.9)	0.969
Approach behaviors (n ≥ 1,%)						
Approach behavior composite score	19 (32.8)	9 (13.6)	0.011	19 (31.7)	10 (15.4)	0.031
Nocturnal hyperactivity	6 (10.3)	1 (1.5)	0.033	6 (10.0)	1 (1.5)	0.040
Eating behavior	6 (10.3)	1 (1.5)	0.033	6 (10.0)	1 (1.5)	0.040
Creativity	5 (8.6)	1 (1.5)	0.066	6 (10.0)	1 (1.5)	0.040
Hobbyism	6 (10.3)	4 (6.1)	0.382	6 (10.0)	5 (7.7)	0.649
Punding	0 (0)	0 (0)	NA	0 (0)	0 (0)	NA
Risk-taking behavior	2 (3.4)	0 (0)	0.128	2 (3.3)	0 (0)	0.138
Compulsive shopping	1 (1.7)	1 (1.5)	0.927	2 (3.3)	1 (1.5)	0.512
Pathological gambling	2 (3.4)	1 (1.5)	0.484	2 (3.3)	1 (1.5)	0.512
Hypersexuality	2 (3.4)	0 (0)	0.128	2 (3.3)	0 (0)	0.138
Excess in motivation	5 (8.8)	0 (0)	0.014	5 (8.5)	0 (0)	0.017

*LPD* Left Parkinson’s Disease, *RPD* Right Parkinson’s Disease, *UPDRS* Unified Parkinson’s Disease Rating Scale, *MDRS* Mattis Dementia Rating Scale, *FAB* Frontal Assessment Battery, *NA* not applicable.

*Of the 128 patients included, 4 patients with indistinguishable hemibody onset and 3 patients with symmetric right and left UPDRS motor subscores were excluded from the corresponding analyses.

Of the 198 de novo PD patients initially enrolled in the “honeymoon” study, we excluded 46 patients receiving IMAO-B, 21 patients taking psychotropic drugs including anxiolytics and/or antidepressants, and 3 patients taking both IMAO-B and psychotropic drugs. Moreover, four patients who reported bilateral side of motor onset and three patients for whom right and left UPDRS part III subscores were equal were ruled out of the corresponding analyses. Finally, six patients classified as RPD according to MDS-UPDRS-III score reported left hemibody disease onset. Conversely, four patients classified as LPD according to MDS-UPDRS-III score reported right hemibody disease onset.

We first examined whether motor symptom laterality, determined from MDS-UPDRS motor score, modulated the approach (AppCS) and avoidance (AvCS) composite scores derived from the ASBPD. RPD patients exhibited significantly lower frequency than LPD patients on AppCS (*p* = 0.031) while RPD and LPD patients did not differ on AvCS. Analyses of score components showed that RPD patients exhibited significantly lower frequency on the ASBPD for nocturnal hyperactivity (*p* = 0.040), eating behavior (*p* = 0.040), creativity (*p* = 0.040) and excess in motivation (*p* = 0.017) than LPD patients. The results of these score component analyses did not survive False Discovery Rate (FDR) correction for multiple comparisons. Statistical findings were similar when using motor symptom laterality based on the side of motor onset, with the exception of creativity.

## Discussion

In this study, we demonstrated that patients with de novo drug-naïve PD had different motivated behavior patterns according to motor sign asymmetry regarding approach behaviors but not avoidance behaviors. These results raise questions about the role of PD asymmetry in motivational imbalance and the occurrence of behavioral disorders in PD.

### Motor sign asymmetry and approach behaviors

For the approach behaviors, we found lower frequency of AppCS score, nocturnal hyperactivity, eating behavior, and excess in motivation in de novo drug-naïve RPD compared to de novo drug-naïve LPD regardless of the method used to assess PD asymmetry. Moreover, we found lower frequency of creativity in de novo drug naïve RPD according to the MDS-UPDRS motor score dichotomy and a trend toward significance when considering the side of motor onset. These results are consistent with previous neuropsychological studies in PD, which highlighted lesser reward sensitivity and approach behaviors in the “OFF-medication” state in RPD compared to LPD, in line with our working hypothesis^31,32^. Interestingly, these studies also emphasized a reversed pattern in reward sensitivity in PD patients in the “ON-medication” state, with greater gain sensitivity in RPD than in LPD^31,32^. These later results are in line with a recent study dedicated to more advanced PD patients receiving pulsatile DRT, which has shown higher eating behavior, creativity, and AppCS scores, and more impulse control disorders (ICD) for RPD compared to LPD^23^. Taken together, all these data are relevant and illustrate the close interaction between frontal lobe lateralization in reward processing, PD asymmetry, and DRT in motivated behavior disorders. We showed that asymmetric motor signs in PD may independently contribute to motivational imbalance, resulting in subtle but clinically significant distinct approach behavior patterns in LPD and RPD, irrespective of disease duration and DRT. In de novo RPD, dopaminergic denervation predominates within the left fronto-striatal circuits, which leads to an imbalance that may disfavor reward processing and approach behaviors. According to both the behavioral sensitization and overdose hypotheses, this imbalance could be reversed in patients with advanced RPD who are taking DRT^23,33–35^. Consequently, RPD patients taking DRT seem to experience an excess of approach behaviors compared to LPD patients taking DRT^23^. Regarding the sensitization hypothesis, non-physiological pulsatile stimulation of dopamine receptors in advanced RPD could favor molecular changes within the more denervated left hemisphere, promoting an exaggerated long-term potentiation, reward processing, and the occurrence of ICD^33^. This is in line with levodopa-induced dyskinesia pathophysiology, which embodies motor sensitization and usually predominates in the most affected side of PD^36^. When considering the dopamine overdose hypothesis, which assumes an inverted U-shaped relationship between dopamine levels and behavioral performances, DRT could restore dopamine level and optimal functioning within the most denervated hemisphere but with a relative “overdosing” effect of the other hemisphere accordingly^34^. In both cases, it could lead, under DRT, to a reverse pattern of motivated behaviors in favor of the most denervated hemisphere^34,35^.

### PD asymmetry and avoidance behaviors

Conflicting results have been reported regarding avoidance behaviors and PD lateralization, with either no difference or opposite patterns in LPD and RPD^19–22,24^. Our results revealed that de novo drug-naïve LPD did not differ from RPD regarding avoidance behaviors, which raises questions about the link between motivational imbalance and these behaviors. Apathy, anxiety and depression have been previously characterized as a reward deficiency syndrome embodied by the so-called neuropsychiatric triad of PD^6,37^. This amotivational syndrome has been linked to combined and widespread dopaminergic and serotonergic denervation within the mesocorticolimbic pathway^4,38,39^. However, some data challenge this view and outline the role of other brain circuits and neurotransmitter systems in apathy, which may involve cognitive functions and executive control in addition to motivation^40,41^. Lastly, anxiety may be considered to be partly independent of reward processing but related to dysfunction within the fear circuit involving the amygdala among other structures^42^.

### Limitations

Even though it is based on a relatively large cohort compared with previous work, our study has some limitations. We did not use striatal dopamine transporter imaging to confirm asymmetric dopaminergic denervation, even though our hypothesis was based on motor sign asymmetry. Moreover, our results related to score components for approach behaviors did not persist after FDR correction for multiple comparisons. Thus, these results, albeit consistent with other data in advanced PD on DRT, should be considered as exploratory and viewed as hypothesis generating.

Finally, we used the ASBPD to consider the behavioral spectrum in PD by dividing these manifestations into avoidance (hypodopaminergic) and approach (hyperdopaminergic) behaviors. Although this division is clinically relevant, it is an oversimplification, since other neurotransmitter systems are also involved in these neuropsychiatric signs^4^.

In summary, our results support the hypothesis that asymmetric motor signs are associated with imbalanced motivated behaviors in de novo drug-naïve PD. If confirmed in larger studies, these results should be taken into account for the personalized choice of dopaminergic treatment and its adjustment over the course of the disease.

## Data Availability

Anonymized data of this study will be available from the corresponding author on reasonable request from any qualified researcher, following the EU General Data Protection Regulation. The study protocol and statistical analysis plan will be shared upon request.

## References

[1] Rodriguez-Oroz MC, Jahanshahi M, Krack P, Litvan I, Macias R (2009). Initial clinical manifestations of Parkinson’s disease: Features and pathophysiological mechanisms. Lancet Neurol..

[2] Weintraub D, Burn DJ (2011). Parkinson’s disease: The quintessential neuropsychiatric disorder. Mov. Disord..

[3] Pont-Sunyer C, Hotter A, Gaig C, Seppi K, Compta Y (2015). The onset of nonmotor symptoms in Parkinson’s disease (the ONSET PD study). Mov. Disord..

[4] Castrioto A, Thobois S, Carnicella S, Maillet A, Krack P (2016). Emotional manifestations of PD: Neurobiological basis. Mov. Disord..

[5] Voon V, Napier TC, Frank MJ, Sgambato-Faure V, Grace AA (2017). Impulse control disorders and levodopa-induced dyskinesias in Parkinson’s disease: An update. Lancet Neurol..

[6] Pagonabarraga J, Kulisevsky J, Strafella AP, Krack P (2015). Apathy in Parkinson’s disease: Clinical features, neural substrates, diagnosis, and treatment. Lancet Neurol..

[7] Reuter-Lorenz P, Davidson RJ (1981). Differential contributions of the two cerebral hemispheres to the perception of happy and sad faces. Neuropsychologia.

[8] Davidson RJ (2004). What does the prefrontal cortex “do” in affect: Perspectives on frontal EEG asymmetry research. Biol. Psychol..

[9] Demaree HA, Everhart DE, Youngstrom EA, Harrison DW (2005). Brain lateralization of emotional processing: Historical roots and a future incorporating “dominance”. Behav. Cogn. Neurosci. Rev..

[10] Ross ED (2021). Differential hemispheric lateralization of emotions and related display behaviors: Emotion-type hypothesis. Brain Sci..

[11] Harciarek M, Mańkowska A (2021). Hemispheric stroke: Mood disorders. Handb. Clin. Neurol..

[12] Cotovio G, Oliveira-Maia AJ (2022). Functional neuroanatomy of mania. Transl. Psychiatry.

[13] Aberg KC, Doell KC, Schwartz S (2015). Hemispheric asymmetries in striatal reward responses relate to approach-avoidance learning and encoding of positive-negative prediction errors in dopaminergic midbrain regions. J. Neurosci..

[14] Irwin DJ, McMillan CT, Xie SX, Rascovsky K, Van Deerlin VM (2018). Asymmetry of post-mortem neuropathology in behavioural-variant frontotemporal dementia. Brain.

[15] Djaldetti R, Ziv I, Melamed E (2006). The mystery of motor asymmetry in Parkinson’s disease. Lancet Neurol..

[16] Riederer P, Jellinger KA, Kolber P, Hipp G, Sian-Hülsmann J (2018). Lateralisation in Parkinson disease. Cell Tissue Res..

[17] Kempster PA, Gibb WR, Stern GM, Lees AJ (1989). Asymmetry of substantia nigra neuronal loss in Parkinson’s disease and its relevance to the mechanism of levodopa related motor fluctuations. J. Neurol. Neurosurg. Psychiatry.

[18] Wang J, Yang QX, Sun X, Vesek J, Mosher Z (2015). MRI evaluation of asymmetry of nigrostriatal damage in the early stage of early-onset Parkinson’s disease. Parkinsonism Relat. Disord..

[19] Pellicano C, Assogna F, Cravello L, Langella R, Caltagirone C (2015). Neuropsychiatric and cognitive symptoms and body side of onset of parkinsonism in unmedicated Parkinson’s disease patients. Parkinsonism Relat. Disord..

[20] Erro R, Pappatà S, Amboni M, Vicidomini C, Longo K (2012). Anxiety is associated with striatal dopamine transporter availability in newly diagnosed untreated Parkinson’s disease patients. Parkinsonism Relat. Disord..

[21] Foster PS, Drago V, Mendez K, Witt JC, Crucian GP (2013). Mood disturbances and cognitive functioning in Parkinson’s disease: The effects of disease duration and side of onset of motor symptoms. J. Clin. Exp. Neuropsychol..

[22] Modestino EJ, Amenechi C, Reinhofer A, O’Toole P (2017). Side-of-onset of Parkinson’s disease in relation to neuropsychological measures. Brain Behav..

[23] Phillipps C, Longato N, Béreau M, Carrière N, Lagha-Boukbiza O (2020). Is motor side onset of Parkinson’s disease a risk factor for developing impulsive-compulsive behavior? A cross-sectional study. Mov. Disord..

[24] Erro R, Santangelo G, Picillo M, Vitale C, Amboni M (2013). Side of onset does not influence cognition in newly diagnosed untreated Parkinson’s disease patients. Parkinsonism Relat. Disord..

[25] Castrioto A, Thobois S, Anheim M, Quesada JL, Lhommée E (2020). A randomized controlled double-blind study of rotigotine on neuropsychiatric symptoms in de novo PD. NPJ Parkinsons Dis..

[26] Voruz P, Pierce J, Ahrweiller K, Haegelen C, Sauleau P (2022). Motor symptom asymmetry predicts non-motor outcome and quality of life following STN DBS in Parkinson’s disease. Sci. Rep..

[27] Foster ER, Black KJ, Antenor-Dorsey JAV, Perlmutter JS, Hershey T (2008). Motor asymmetry and substantia nigra volume are related to spatial delayed response performance in Parkinson disease. Brain Cogn..

[28] Rieu I, Martinez-Martin P, Pereira B, De Chazeron I, Verhagen Metman L (2015). International validation of a behavioral scale in Parkinson’s disease without dementia. Mov. Disord..

[29] Ardouin C, Chéreau I, Llorca P-M, Lhommée E, Durif F (2009). Assessment of hyper- and hypodopaminergic behaviors in Parkinson’s disease. Rev. Neurol. (Paris).

[30] Béreau M, Fleury V, Bouthour W, Castrioto A, Lhommée E (2018). Hyperdopaminergic behavioral spectrum in Parkinson’s disease: A review. Rev. Neurol. (Paris).

[31] Maril S, Hassin-Baer S, Cohen OS, Tomer R (2013). Effects of asymmetric dopamine depletion on sensitivity to rewarding and aversive stimuli in Parkinson’s disease. Neuropsychologia.

[32] Porat O, Hassin-Baer S, Cohen OS, Markus A, Tomer R (2014). Asymmetric dopamine loss differentially affects effort to maximize gain or minimize loss. Cortex.

[33] Castrioto A, Carnicella S, Fraix V, Chabardes S, Moro E (2017). Reversing dopaminergic sensitization. Mov. Disord..

[34] Vaillancourt DE, Schonfeld D, Kwak Y, Bohnen NI, Seidler R (2013). Dopamine overdose hypothesis: Evidence and clinical implications. Mov. Disord..

[35] Meder D, Herz DM, Rowe JB, Lehéricy S, Siebner HR (2019). The role of dopamine in the brain - lessons learned from Parkinson’s disease. Neuroimage.

[36] Bastide MF, Meissner WG, Picconi B, Fasano S, Fernagut P-O (2015). Pathophysiology of L-dopa-induced motor and non-motor complications in Parkinson’s disease. Prog. Neurobiol..

[37] Béreau M, Castrioto A, Lhommée E, Maillet A, Gérazime A (2022). Fatigue in de novo Parkinson’s disease: Expanding the Neuropsychiatric Triad?. J. Parkinsons Dis..

[38] Maillet A, Krack P, Lhommée E, Météreau E, Klinger H (2016). The prominent role of serotonergic degeneration in apathy, anxiety and depression in de novo Parkinson’s disease. Brain.

[39] Maillet A, Météreau E, Tremblay L, Favre E, Klinger H (2021). Serotonergic and dopaminergic lesions underlying Parkinsonian neuropsychiatric signs. Mov. Disord..

[40] Béreau M, Kibleur A, Servant M, Clément G, Dujardin K (2023). Motivational and cognitive predictors of apathy after subthalamic nucleus stimulation in Parkinson’s disease. Brain.

[41] Béreau M, Van Waes V, Servant M, Magnin E, Tatu L (2023). Apathy in Parkinson’s disease: Clinical patterns and neurobiological basis. Cells.

[42] Carey G, Görmezoğlu M, de Jong JJA, Hofman PAM, Backes WH (2021). Neuroimaging of anxiety in Parkinson’s disease: A systematic review. Mov. Disord..

